# Cardiac Magnetic Resonance Imaging Right Ventricular Longitudinal Strain Predicts Mortality in Patients Undergoing TAVI

**DOI:** 10.3389/fcvm.2021.644500

**Published:** 2021-05-07

**Authors:** Johannes Schmid, Claus Kamml, David Zweiker, Dominik Hatz, Albrecht Schmidt, Ursula Reiter, Gabor G. Toth, Michael Fuchsjäger, Andreas Zirlik, Josepha S. Binder, Peter P. Rainer

**Affiliations:** ^1^Division of General Radiology, Department of Radiology, Medical University of Graz, Graz, Austria; ^2^Division of Cardiology, Department of Internal Medicine, Medical University of Graz, Graz, Austria; ^3^Third Medical Department of Cardiology and Intensive Care, Wilhelminenhospital, Vienna, Austria; ^4^BioTechMed Graz, Graz, Austria

**Keywords:** transcatheter aortic valve implantation, aortic valve stenosis, magnetic resonance imaging, MRI, right ventricular function, strain, survival analysis, mortality

## Abstract

**Background:** Right ventricular (RV) function predicts survival in numerous cardiac conditions, including left heart disease. The reference standard for non-invasive assessment of RV function is cardiac magnetic resonance imaging (CMR). The aim of this study was to investigate the association between pre-procedural CMR-derived RV functional parameters and mortality in patients undergoing transcatheter aortic valve implantation (TAVI).

**Methods:** Patients scheduled for TAVI were recruited to undergo pre-procedural CMR. Volumetric function and global longitudinal and circumferential strain (GLS and GCS) of the RV and left ventricle (LV) were measured. The association with the primary endpoint (1-year all-cause mortality) was analyzed with Cox regression.

**Results:** Of 133 patients undergoing CMR, 113 patients were included in the analysis. Mean age was 81.8 ± 5.8 years, and 65% were female. Median follow-up was 3.9 [IQR 2.3–4.7] years. All-cause and cardiovascular mortality was 14 and 12% at 1 year, and 28 and 20% at 3 years, respectively. One-year all-cause mortality was significantly predicted by RV GLS [HR = 1.109 (95% CI: 1.023–1.203); *p* = 0.012], RV ejection fraction [HR = 0.956 (95% CI: 0.929–0.985); *p* = 0.003], RV end-diastolic volume index [HR = 1.009 (95% CI: 1.001–1.018); *p* = 0.025], and RV end-systolic volume index [HR = 1.010 (95% CI: 1.003–1.017); *p* = 0.005]. In receiver operating characteristic (ROC) analysis for 1-year all-cause mortality, the area under the curve was 0.705 (RV GLS) and 0.673 (RV EF). Associations decreased in strength at longer follow-up. None of the LV parameters was associated with mortality.

**Conclusions:** RV function predicts intermediate-term mortality in TAVI patients while LV parameters were not associated with outcomes. Inclusion of easily obtainable RV GLS may improve future risk scores.

## Introduction

Transcatheter aortic valve implantation (TAVI) is a minimally invasive treatment for patients suffering from severe aortic stenosis and is now available for more than a decade. Since then, procedure numbers are steadily increasing and are expected to rise further (1). While initially reserved for patients with a high-risk profile for surgical valve replacement, indications have been extended to intermediate-risk patients and trials already showed promising intermediate-term results in low-risk groups (2, 3). Predicting the outcome of patients undergoing TAVI is challenging as the commonly applied clinical risk scores perform only moderately (4, 5). Thus, adequate surrogate markers to improve outcome prediction are needed.

Until recently, the right ventricle (RV) has not received much attention in this respect. However, emerging evidence shows that the RV plays a key role in many cardiac conditions, including primary left heart disease (6). A prognostic value of RV function has not only been demonstrated in pulmonary hypertension (7) but also in heart failure with preserved and reduced ejection fraction (EF) (8, 9), dilated cardiomyopathy (10), or myocardial infarction (11). Echocardiographic studies indicate that RV dysfunction is frequent in patients with severe aortic stenosis and is associated with mortality (12–15).

Due to the complex shape of the RV, standard echocardiography relies on tricuspid annular systolic excursion, fractional area change, or more recently strain as surrogates for RV function. Unlike echocardiography, cardiac magnetic resonance imaging (CMR) not only is capable of myocardial tissue characterization but also allows for more accurate measurement of cardiac chamber volumes. Measures of myocardial deformation derived from CMR feature tracking predict cardiovascular events (16). CMR is therefore regarded the reference standard for non-invasive assessment of RV volumes and function (17).

This study aims to elucidate the association between pre-procedural RV functional parameters derived from CMR and mortality in patients undergoing TAVI.

## Materials and Methods

In this single-center cohort study, patients with severe aortic stenosis scheduled for TAVI at the Division of Cardiology, Medical University of Graz were prospectively recruited to undergo pre-procedural CMR between May 2011 and March 2015, in the absence of CMR contraindications (incompatible metal implants such as pacemakers, severely reduced kidney function, claustrophobia). Patients who underwent a successful transfemoral TAVI procedure with a CoreValve (Medtronic) prosthesis were included in the study. Cases with a CMR image quality insufficient to allow reliable analysis were excluded.

The primary endpoint was all-cause mortality (18) at 1 year, which corresponds to the recommended minimum life expectancy for patients eligible for TAVI (19). Additionally, cardiovascular mortality was defined according to VARC-2 criteria (20) and served as a secondary endpoint. After a follow-up of at least 3 years, mortality data were gathered via review of medical records, via phone contact, or by request at the national death register. All patients gave written informed consent. The study was approved by the ethics committee of the Medical University of Graz (No. 25-437ex12/13) and complies with the Declaration of Helsinki.

### Cardiac Magnetic Resonance Imaging

CMR was performed on a 1.5-Tesla scanner (Magnetom Sonata, Siemens Healthcare). Steady-state free precession cine sequences with retrospective electrocardiographic gating were acquired during free breathing in two-, three-, and four-chamber views and a stack of gapless slices in short-axis view to cover the entire left and right ventricle. Typical protocol parameters were: echo time 1.2 ms; measured temporal resolution 37–51 ms reconstructed to 30 phases; resolution 1.4 × 1.7–2.0 × 6 mm^3^ for long axes and 1.4 × 2.2 × 8 mm^3^ for short-axis views. To compensate for breathing artifacts, threefold averaging was used. Using dedicated software (cvi42, Version 5.6.5, Circle Cardiovascular Imaging), right and left ventricular (LV) end-diastolic and end-systolic volumes (EDV, ESV) were determined from a short-axis stack according to recommendations (21) and indexed to body surface area. Papillary muscles were included in the volumes. Global longitudinal and circumferential strains (GLS and GCS) were measured in both ventricles by means of feature tracking using the 2D model. RV free wall GLS and LV GLS were measured from a four-chamber slice. For GCS, the most basal and apical slices were rejected if tracking was inappropriate (assessed visually).

### Statistics

Statistical analysis was performed using SPSS Statistics 25.0.0.1 (IBM). Parameters are presented as mean ± standard deviation, median [interquartile range] or percentage (absolute numbers). The primary endpoint was 1-year all-cause mortality; associations with mortality were analyzed in uni- and multivariate Cox regression models. To ensure acceptable proportional hazards in Cox regression, follow-up was truncated at 1 year. For Kaplan–Meier plots, variables were dichotomized at their median. Receiver operating characteristic (ROC) analysis was performed for 1-year all-cause mortality. Area under the curve was compared with the DeLong method using the package pROC in R 3.5.1 (The R Foundation for Statistical Computing). A *p* value of <0.05 was considered statistically significant.

## Results

### Study Population

**Figure 1:**
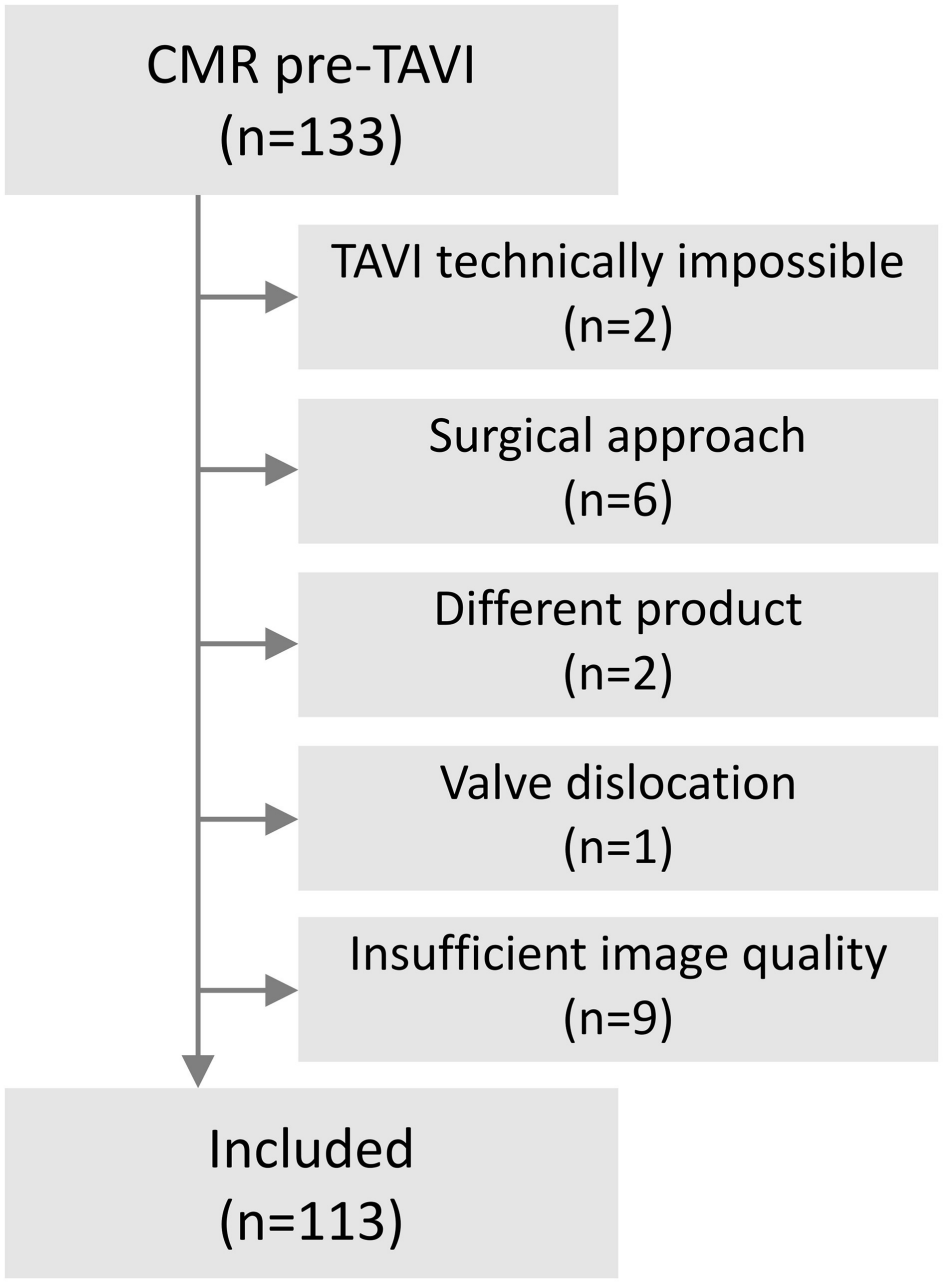
Flow chart of included patients.

During the study period, a total of 296 patients underwent TAVI at our institution; of those, 133 patients had pre-procedural CMR (at a median of 2 [1–4] days before the procedure). Exclusion criteria were met by 20 patients, leaving 113 patients qualifying for analysis (Figure 1). The cohort comprised elderly patients with a mean age of 81.8 ± 5.8 years and a slight female predominance of 65%. Most of the patients suffered from high gradient severe aortic stenosis (81%); for the detailed distribution of types of aortic stenosis, see Supplementary Figure 1 (19). The majority (80%) of the cohort underwent pre-procedural right heart catheterization. More than half (56%) of the patients suffered from pulmonary hypertension, defined as mPAP >25 mmHg, and even 74% if applying the recent 20 mm Hg definition (22) (details in Supplementary Figure 2). RV enlargement (elevated EDV index) was found in 8.8% (*n* = 10) and an enlarged LV in 32.7% (*n* = 37) (23). RV and LV EF was <50% in 28.3% (*n* = 32) and 32.7% (*n* = 37), respectively. Detailed baseline characteristics are presented in Table 1.

**Table 1:** Baseline characteristics according to 1-year mortality.

	**All patients (*n* = 113)**	**Survived 1st year (*n* = 97)**	**Died in 1st year (*n* = 16)**	***p*-value**
Age, years	81.8 ± 5.8	81.4 ± 5.9	84.2 ± 5.0	0.071
Female sex	65% (73)	64.9% (63)	62.5% (10)	0.529
Body mass index	26.0 ± 4.8	26.3 ± 4.3	24.3 ± 6.9	0.280
CAD	78% (88)	75% (73)	94% (15)	0.117
CABG or PCI	38% (43)	38% (37)	38% (6)	0.961
PAD	27% (31)	25% (24)	44% (7)	0.114
Diabetes	26% (29)	25% (24)	31% (5)	0.552
Hypertension	83% (94)	84% (81)	81% (13)	0.731
Atrial fibrillation	41% (46)	38% (37)	56% (9)	0.172
NT-proBNP, ng/L	1,722 [654–3,515]	1,583 [624–3,515]	2,701 [2,152–3,871]	0.025^*^
Troponin T, ng/L	22 [12–38]	21 [12–38]	25 [16–65]	0.218
eGFR, ml/min/1.73 m^2^	56.4 ± 16.9	57.1 ± 17.6	52.2 ± 11.0	0.280
AVA, cm^2^	0.67 ± 0.17	0.67 ± 0.16	0.68 ± 0.19	0.832
STS score (%)	3.30 [2.55–5.33]	3.14 [2.48–5.18]	5.15 [3.83–6.65]	0.002^*^
EUROscore II (%)	4.77 [3.11–8.05]	4.77 [3.05–7.52]	5.94 [4.27–10.54]	0.096
mPAP, mmHg	29.4 ± 11.2	29.6 ± 11.7	28.5 ± 7.9	0.747
mPAP >25 mmHg	56.0% (51/91)	54.5% (42/77)	64.3% (9/14)	0.354
PCWP, mmHg	18.0 ± 8.3	18.1 ± 8.5	17.1 ± 7.4	0.679
**CMR parameters**				
RV-EF, %	54.7 ± 12.8	56.2 ± 11.1	45.5 ± 18.4	0.007^*^
RV-EDV, ml	131.5 [107.3–161.1]	131.5 [105.3–159.7]	134.0 [113.4–171.3]	0.168
RV-EDV index, ml/m^2^	74.6 [63.3–92.5]	73.0 [62.0–91.8]	80.3 [65.8–97.6]	0.042^*^
RV-ESV, ml	59.6 [43.0–72.8]	55.7 [42.5–71.44]	67.2 [46.8–106.6]	0.023^*^
RV-ESV index, ml/m^2^	33.2 [24.5–42.3]	32.1 [23.8–40.5]	40.1 [28.7–62.4]	0.059
RV-GLS, %	−21.3 ± 5.8	−21.9 ± 5.6	−17.7 ± 5.8	0.007^*^
RV-GCS, %	−13.5 ± 3.6	13.7 ± 3.5	−12.3 ± 4.5	0.158
LV-EF, %	52.5 ± 13.0	53.0 ± 11.9	49.5 ± 18.3	0.588
LV-EDV, ml	154.2 [117.1–198.3]	155.2 [118.6–198.1]	146.2 [114.8–214.8]	0.827
LV-EDV index, ml/m^2^	88.7 [70.6–107.8]	90.0 [69.9–105.22]	81.1 [73.4–123.6]	0.446
LV-ESV, ml	69.7 [45.3–101.1]	69.8 [45.3–100.4]	60.1 [44.7–131.1]	0.726
LV-ESV index, ml/m^2^	39.6 [27.4–54.1]	39.6 [27.4–54.0]	35.6 [28.2–71.7]	0.441
LV-GLS, %	−13.2 ± 3.9	−13.4 ± 3.8	−12.1 ± 4.8	0.227
LV-GCS, %	−17.2 ± 4.8	−17.4 ± 4.4	−16.2 ± 6.5	0.361
LV-GRS, %	30.3 ± 11.5	28.4 ± 15.1	30.0 ± 12.0	0.566
LV mass index, g/m^2^	76.5 ± 18.2	76.8 ± 18.1	74.7 ± 19.3	0.675

*Values are mean ± standard deviation, median [interquartile range] or percentage (absolute numbers)*.

*CAD, coronary artery disease; CABG, coronary artery bypass graft; PCI, percutaneous coronary intervention; PAD, peripheral artery disease; NT-proBNP, N-terminal pro-brain natriuretic peptide; eGFR, estimated glomerular filtration rate; AVA, aortic valve area (from echocardiography); mPAP, mean pulmonary artery pressure; PCWP, pulmonary capillary wedge pressure; RV, right ventricle; LV, left ventricle; EF, ejection fraction; EDV, end-diastolic volume; ESV, end-systolic volume; GLS, global longitudinal strain; GCS, global circumferential strain; GRS, global radial strain; mPAP and PCWP from right heart catheterization (n = 91)*.

*p-values from Student's t-test or Fisher's exact test; variables transformed (logarithm or square root) if necessary*.

* *p < 0.05*.

### Survival Analysis

**Figure 2:**
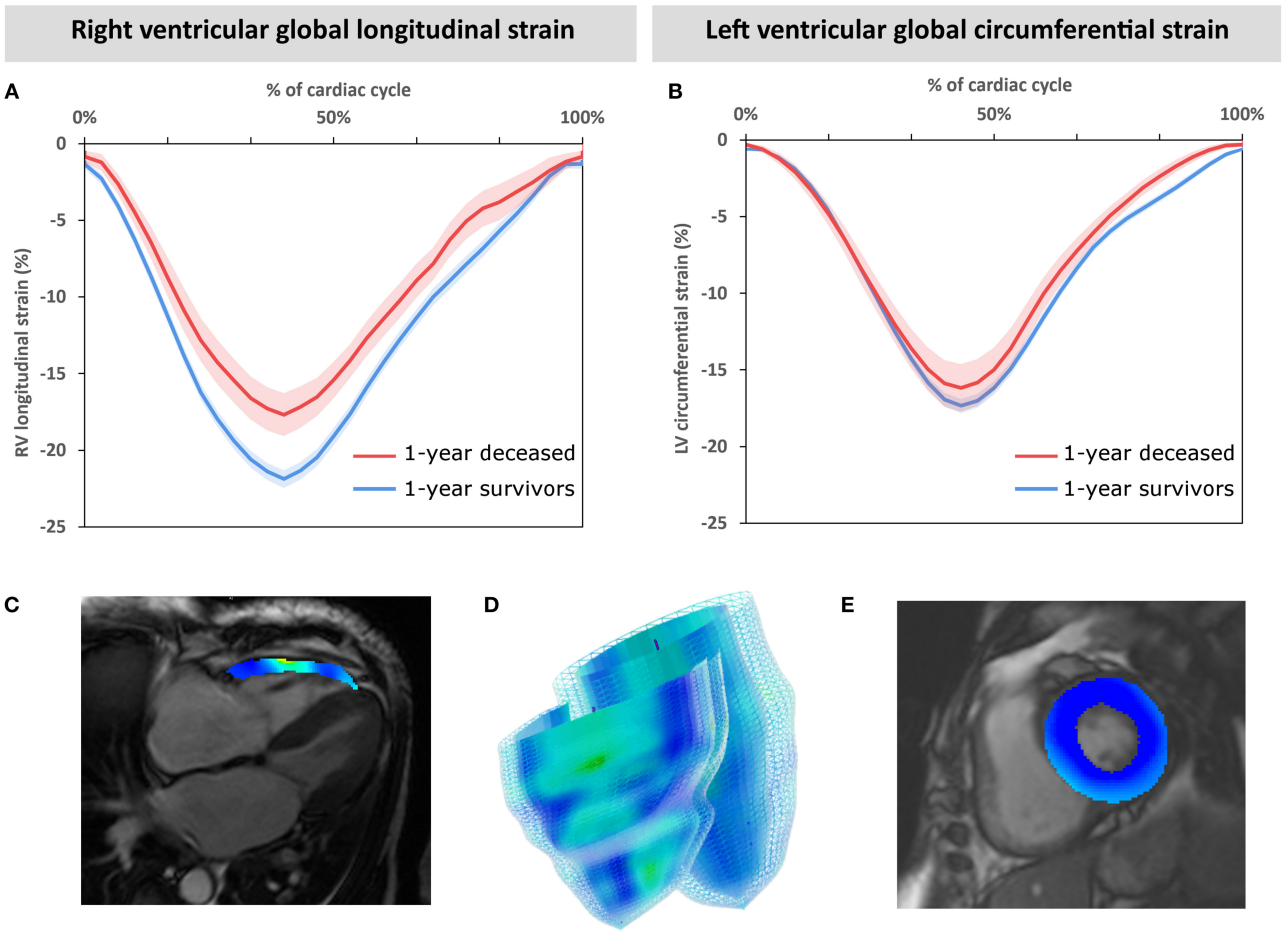
Mean global strain curves of the cohort grouped by 1-year mortality. Individual global strain curves of the entire cohort are aligned at peak systolic strain and averaged in groups according to 1-year mortality. Mean and standard error of the mean are displayed for right ventricular longitudinal strain **(A)** and left ventricular circumferential strain **(B)**. Exemplary cardiac magnetic resonance images in end-systole with color-coded strain overlay **(C, E)** and a 3D model **(D)** are displayed.

Patients were followed up for a median of 3.9 [2.3–4.7] years. Three-year follow-up was completed by all survivors. All-cause mortality was 14% (*n* = 16) and 28% (*n* = 32), and cardiovascular mortality was 12% (*n* = 13) and 20% (*n* = 23) at 1 and 3 years, respectively. Significant differences in RV EF, RV GLS, RV ESV, and RV EDV index were found between those who survived after 1 year and those who did not (Table 1, Figure 2). We also observed higher N-terminal pro-brain natriuretic peptide values in patients who did not survive 1 year. LV imaging parameters, however, did not differ between survivors and non-survivors. There was no significant difference in mortality between male and female patients.

**Figure 3:**
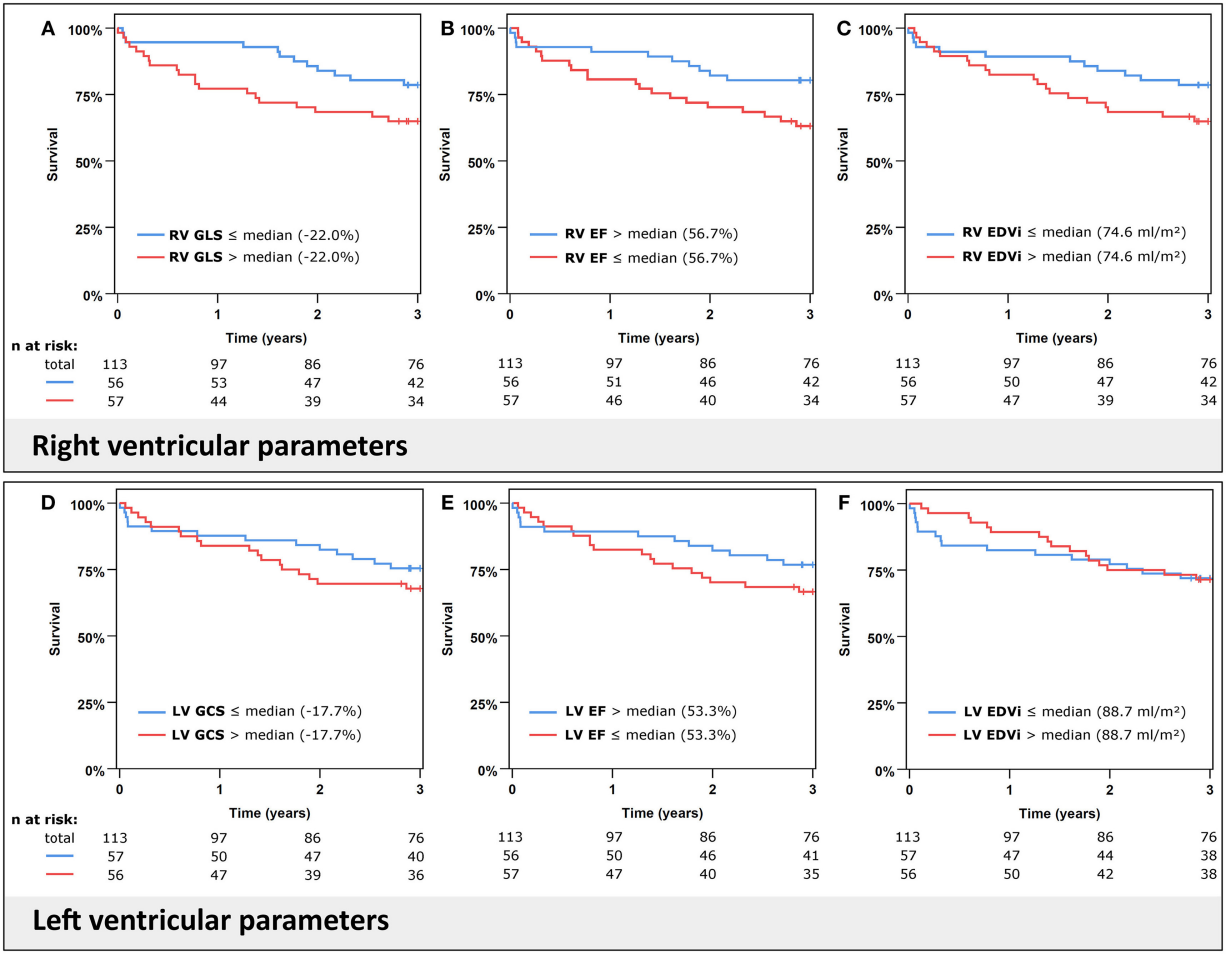
Kaplan–Meier plots for all-cause mortality. Cumulative survival according to right ventricular (RV) global longitudinal strain (GLS) **(A)**, ejection fraction (EF) **(B)**, end-diastolic volume index (EDVi) **(C)** in the upper panels and left ventricular (LV) global circumferential strain (GCS) **(D)**, EF **(E)**, and EDVi **(F)** in the lower panels. Variables are stratified by their median.

Kaplan–Meier analyses revealed the impact of RV parameters on mortality especially during the first years of follow-up. This association was partially attenuated at extended follow-up (Supplementary Figure 4). To account for the non-proportional behavior of survival curves at long-term follow-up and better reflect intermediate-term outcomes, follow-up was truncated at 1 year for Cox regression analyses. Results from univariate Cox regressions of RV and LV parameters are given in Table 2 and illustrated by Kaplan–Meier plots in Figure 3. RV GLS, RV EF, and RV volumes were significantly associated with 1-year all-cause mortality. In contrast to RV function, the corresponding LV parameters did not significantly predict mortality in the cohort.

**Table 2:** Univariate Cox regression of right and left ventricular parameters for 1-year all-cause mortality.

	**HR (95% CI)**	***p*-value**
**Right ventricle**		
RV GLS (%)	1.109 (1.023–1.203)	0.012^*^
RV GCS (%)	1.099 (0.960–1.259)	0.169
RV EF (%)	0.956 (0.929–0.985)	0.003^*^
RV EDVi (ml/m^2^)	1.009 (1.001–1.018)	0.025^*^
RV ESVi (ml/m^2^)	1.010 (1.003–1.017)	0.005^*^
**Left ventricle**		
LV GLS (%)	1.073 (0.952–1.210)	0.245
LV GCS (%)	1.041 (0.942–1.150)	0.430
LV EF (%)	0.984 (0.950–1.020)	0.378
LV EDVi (ml/m^2^)	1.008 (0.991–1.026)	0.365
LV ESVi (ml/m^2^)	1.012 (0.996–1.028)	0.149
LV mass index (g/m^2^)	0.993 (0.967–1.021)	0.633
**Right heart catheterization (*****n*** **=** **91)**		
mPAP > 25 mmHg	1.40 (0.47–4.18)	0.547
**Right ventricular–pulmonary artery coupling (*****n*** **=** **91)**		
RV GLS/mPAP	1.26 (0.44–3.64)	0.666

*EF, ejection fraction; GLS, global longitudinal strain; GCS, global circumferential strain; EDVi, end-diastolic volume index; ESVi, end-systolic volume index; mPAP, mean pulmonary artery pressure*.

* *p < 0.05*.

We additionally performed several multivariate Cox regression models with RV GLS or RV EF adjusted for the relevant covariates STS score and NT-proBNP (Table 3). RV GLS and RV EF remained significant predictors of 1-year all-cause mortality independent of STS score; however, in models containing NT-proBNP, RV parameters did not reach significance.

**Table 3:** RV GLS in multivariate Cox regressions for 1-year all-cause mortality.

	**HR (95% CI)**	***p*-value**
**Model 1**		
RV GLS (%)	1.094 (1.006–1.189)	0.035^*^
STS score (%)	1.164 (1.046–1.296)	0.005^*^
**Model 2**		
RV GLS (%)	1.076 (0.977–1.186)	0.136
NT-proBNP (log, ng/L)	1.911 (0.549–6.647)	0.309
**Model 3**		
RV EF (%)	0.960 (0.931–0.990)	0.010^*^
STS score (%)	1.170 (1.048–1.305)	0.005^*^
**Model 4**		
RV EF (%)	0.966 (0.929–1.003)	0.074
NT-proBNP (log, ng/L)	1.604 (0.453–5.675)	0.464

*RV, right ventricle; GLS, global longitudinal strain; EF, ejection fraction*.

* *p < 0.05*.

On analysis of the secondary endpoints (Supplementary Table 2), RV EF and RV volumes significantly predicted 1-year and 3-year cardiovascular mortality as well as 3-year all-cause mortality, while RV GLS (though significant for 1-year cardiovascular mortality) just missed the significance level for 3-year all-cause and cardiovascular mortality (*p* = 0.053 and *p* = 0.087).

We also assessed RV to pulmonary artery coupling using the ratio of RV GLS/invasive mean pulmonary artery pressure (mPAP), which was not significantly associated with mortality (Table 2). Interestingly, a modifying effect of elevated mPAP on RV GLS mortality prediction became apparent after 1 year of follow-up (Supplementary Figure 5). The survival curve of patients in the group with better (more negative) RV GLS but with elevated mPAP initially almost paralleled the curve of low mPAP patients during the first year, but then showed markedly increased mortality and aligned with the less negative RV GLS group.

**Figure 4:**
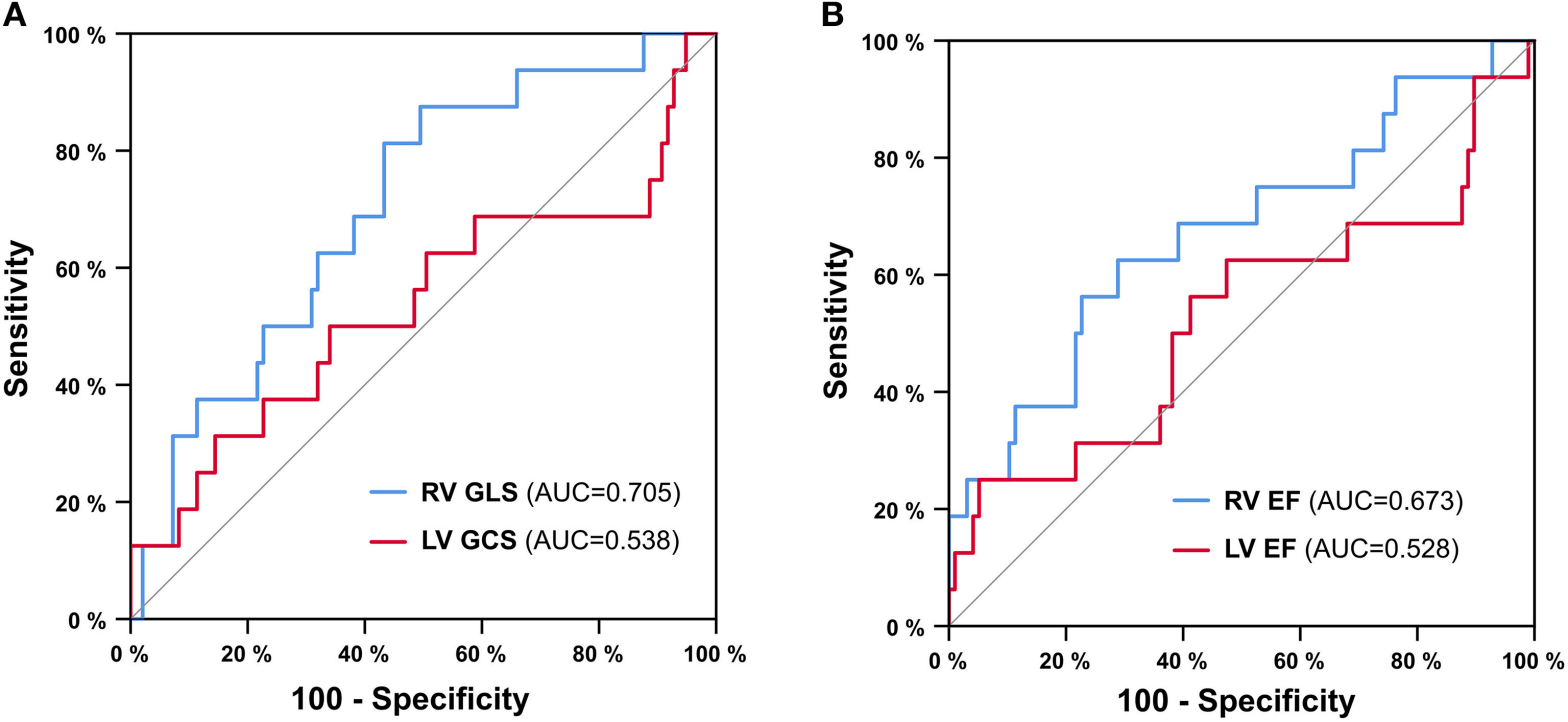
ROC analysis for 1-year all-cause mortality. Right ventricular global longitudinal strain (RV GLS) vs. left ventricular global circumferential strain (LV GCS) **(A)** and right ventricular ejection fraction (RV EF) vs. left ventricular ejection fraction (LV EF) **(B)**. AUC = area under the curve.

Additional ROC analyses for 1-year all-cause mortality (Figure 4) illustrate a better diagnostic accuracy (area under the curve) of RV GLS and RV EF when compared to left ventricular parameters (RV GLS vs. LV GCS: ΔAUC = 0.167, *p* = 0.003; RV EF vs. LV EF: ΔAUC = 0.145, *p* = 0.035). The difference in the area under the curve was not significant for comparisons of RV GLS with RV EF (ΔAUC = 0.032, *p* = 0.663), STS score (ΔAUC = −0.015, *p* = 0.860), or NT-proBNP (ΔAUC = 0.044, *p* = 0.522).

## Discussion

Our data illustrate that RV functional parameters, especially RV GLS and RV EF, associate with intermediate-term outcomes in patients undergoing TAVI. Intriguingly, this association was much stronger than in corresponding LV parameters, which did not predict outcomes in our cohort.

When assessing the RV, the main advantage of CMR is its unique ability to accurately measure volumes, a task that is not easily achieved by echocardiography. Our study shows that both volumetric parameters (such as EF and EDV), as well as measures of RV myocardial contraction (RV GLS), predict mortality after TAVI. These associations were independent from multiparametric clinical risk scores. In contrast to the LV, longitudinal contraction pre-dominates RV function. Thus, GLS reflects RV function better than GCS, which was not associated with outcomes. Compared to volumetric parameters, RV GLS is easy to measure and, importantly, it can also be assessed by widely available echocardiography if acoustic windows permit. In line with our results, RV function assessed by echocardiography is also associated with mortality in patients with severe aortic stenosis (12, 24) and in patients undergoing TAVI (14, 25). Our study confirms these findings in prospectively enrolled patients using CMR as a reference standard.

Importantly, LV function, which is usually central in clinical assessment, did not predict mortality in our cohort. Consistent with our findings, other studies showed that LV EF predicted outcomes only in the subgroup of low gradient aortic stenosis (26, 27). In another large cohort study LV EF did predict 3-year mortality, but only in univariate analysis (28). One aspect that may contribute to the weak predictive power of LV function may be the differential response of the left ventricle with either concentric or eccentric remodeling or hypertrophy. Concentric geometry causes a reduction of ESV and thus tends to increase EF, which is based on the ratio of (EDV-ESV)/EDV. This mechanism may explain the complex and apparently non-linear association between LV function and survival with a reduced early survival of patients in the highest LV EF quartile observed in our cohort, as concentric phenotypes are associated with worse outcomes (29). Similarly, LV GCS or GLS is affected by different types of remodeling that may result in a more complex relationship with outcomes. Previous studies on the predictive ability of LV strains in TAVI patients yielded mixed results (30, 31). Though a high proportion of patients had increased LV mass index, this parameter was not significantly associated with 1-year mortality, similar to results of other large contemporary cohorts undergoing TAVI (32).

When assessing the prognostic value of pulmonary hypertension, we found that the mortality prediction of mPAP became only evident at longer follow-up. To further investigate the interdependency with the pulmonary vasculature, we related RV function assessed by CMR RV GLS to RV afterload using invasively measured mPAP and thereby assessed RV to pulmonary artery coupling. This approach was similarly applied before in other collectives but with estimating rather than directly measuring pulmonary artery pressures and relying on echocardiographic TAPSE (33, 34). Patients in the best (most negative) quartile of the coupling variable (RV GLS/mPAP) had markedly better survival (not shown). In patients with preserved RV GLS, those with pulmonary hypertension (mPAP >25 mmHg) initially had similar survival to those without, but, after 1-year survival, dropped to align with patients with reduced RV GLS. These data suggest that prognosis is worst if RV contractility impairment is already present at baseline with a delayed effect of pulmonary hypertension that precedes manifest RV GLS reduction. Patients with both preserved RV GLS and absent pulmonary hypertension had an excellent prognosis and 3-year survival reached 92%.

When analyzing the survival curves of the full available follow-up, long-term follow-up for more than 3 to 4 years increasingly attenuated survival prediction. This observation is likely explained by the age distribution of TAVI patients where non-procedure-related mortality is high and dominates after a few years.

### Strengths and Limitations

This is the first study to comprehensively analyze the RV with gold standard CMR in patients undergoing TAVI including long-term follow-up and invasive hemodynamic assessment. This was accomplished in a reasonably sized cohort, although larger numbers might have allowed more extensive analyses with higher power for the detection of weaker associations. Our cohort reflects a typical TAVI cohort and mostly consisted of patients with high gradient aortic stenosis; thus, findings may be different in patients suffering from low gradient aortic stenosis where LV function may discriminate outcome better. The use of CMR (excluding patients with pacemakers and severely reduced kidney function) and the non-consecutive nature of our cohort may induce a certain selection bias in the study cohort.

### Conclusion and Outlook

RV function predicts intermediate-term mortality after TAVI while LV-derived parameters do not. In particular, RV GLS is a promising parameter to stratify outcomes after TAVI, as the echocardiographic equivalent measures of longitudinal contraction (GLS and TAPSE) can be easily obtained and included in future prospectively validated clinical risk scores, which may help to improve patient selection.

## Data Availability Statement

The raw data supporting the conclusions of this article will be made available by the authors on request, without undue reservation.

## Ethics Statement

The study involving human participants was reviewed and approved by the Ethics committee of the Medical University of Graz, Graz, Austria. The patients provided their written informed consent to participate in this study.

## Author Contributions

JS and PR conceptualized and designed the study. JS organized and collected data, performed statistical analyses and drafted the manuscript. CK analyzed cardiac magnetic resonance images. DZ and DH collected clinical and outcome data. UR acquired cardiac magnetic resonance images and wrote sections of the manuscript. JB recruited patients. PR, DZ, UR, GT, JB, AZ, MF, and AS revised the manuscript critically for important intellectual content. JS, JB, and PR supervised the study. All authors read and approved the submitted version.

## Conflict of Interest

GT received consultancy fees and unrestricted research support from Abbott, Medtronic, Biotronic and Boston Scientific, not related to this work. The remaining authors declare that the research was conducted in the absence of any commercial or financial relationships that could be construed as a potential conflict of interest.
