# Supplementary material for: Cardiac Magnetic Resonance Imaging Right Ventricular Longitudinal Strain Predicts Mortality in Patients Undergoing TAVI
Source: Front Cardiovasc Med. 2021 May 7;8:644500. doi: 10.3389/fcvm.2021.644500 (PMC8137844; doi:10.3389/fcvm.2021.644500)
Supplement: Supplementary file 1 [file Data_Sheet_1.PDF]

Supplementary Material

1 Supplementary Tables

Supplementary Table 1. Comparison of included patients with all consecutive patients undergoing TAVI within the study's timeframe.

	**Included n=113**	**Not included n=183**	**All consecutive n= 296**	**p-value**
Age, years	81.8 ± 5.8	82.5±5.7	82.1±5.8	0.078
Female sex	64.6% (73)	59.6% (109)	61.5% (182)	0.387
Body mass index	26.0±4.8	26.2±4.3	26.1±4.5	0.622
CAD	77.9% (88)	57.9% (106)	65.5% (194)	<0.001*
CABG or PCI	38.1% (43)	30.6% (56)	33.4% (99)	0.187
PAD	27.4% (31)	14.2% (26)	19.3% (57)	0.005*
Diabetes	25.7% (29)	26.8% (49)	26.4% (78)	0.833
Hypertension	83.2% (94)	81.4% (149)	82.1% (243)	0.700
Atrial fibrillation	40.7% (46)	43.2% (79)	42.2% (125)	0.677
Pacemaker	0% (0)	1.6% (3)	1.0% (3)	0.290
AVA, cm^2	0.67 ± 0.17	0.68±0.19	0.68±0.20	0.454
STS score (%)	3.3 [2.6-5.3]	4.0 [2.7-5.7]	3.7 [2.6-5.4]	0.244
EUROscore II (%)	4.8 [3.1-8.0]	5.0 [3.4-8.7]	5.0 [3.3-8.3]	0.624
mPAP, mmHg	29.4±11.2	31.2±11.9	30.5±11.6	0.255
PCWP, mmHg	18.0±8.3	19.2±9.3	18.7±8.9	0.305

Values are mean ± standard deviation, median [interquartile range] or percentage (absolute numbers).
CAD = coronary artery disease; CABG = coronary artery bypass graft; PCI = percutaneous coronary intervention; PAD = peripheral artery disease; AVA = aortic valve area; mPAP = mean pulmonary artery pressure; PCWP = pulmonary capillary wedge pressure; mPAP and PCWP from right heart catheterization (total n=219).
P-values from Student's t-test, χ^2 test or Fisher's exact test. Variables transformed (logarithm) if necessary. *p<0.05

Supplementary Table 2. Univariate Cox regression for secondary endpoints 3-year all-cause (AC) mortality and 1- and 3-year cardiovascular (CV) mortality.

	**1-year CV mortality**		**3-year AC mortality**		**3-year CV mortality**	
	**HR (95% CI)**	**p-value**	**HR (95% CI)**	**p-value**	**HR (95% CI)**	**p-value**
**Right ventricle**						
RV GLS (%)	1.107 (1.012-1.212)	0.026*	1.059 (0.999-1.122)	0.053	1.061 (0.991-1.136)	0.087
RV GCS (%)	1.151 (0.993-1.335)	0.062	1.045 (0.947-1.154)	0.380	1.105 (0.985-1.240)	0.089
RV EF (%)	0.951 (0.921-0.981)	0.001*	0.970 (0.946-0.994)	0.015*	0.960 (0.934-0.986)	0.003*
RV EDVi (ml/m²)	1.011 (1.003-1.019)	0.010*	1.009 (1.002-1.017)	0.013*	1.010 (1.002-1.018)	0.011*
RV ESVi (ml/m²)	1.011 (1.004-1.018)	0.002*	1.010 (1.003-1.017)	0.003*	1.011 (1.004-1.018)	0.001*
**Left ventricle**						
LV GLS (%)	1.096 (0.962-1.249)	0.167	1.039 (0.952-1.134)	0.392	1.084 (0.981-1.198)	0.115
LV GCS (%)	1.048 (0.940-1.168)	0.401	1.032 (0.958-1.111)	0.408	1.039 (0.954-1.132)	0.380
LV EF (%)	0.982 (0.944-1.021)	0.353	0.989 (0.963-1.016)	0.420	0.986 (0.956-1.016)	0.354
LV EDVi (ml/m²)	1.010 (0.990-1.029)	0.334	1.009 (0.996-1.022)	0.164	1.008 (0.993-1.023)	0.319
LV ESVi (ml/m²)	1.014 (0.997-1.031)	0.112	1.011 (0.998-1.024)	0.106	1.012 (0.998-1.027)	0.102
LV mass index (g/m²)	0.997 (0.967-1.027)	0.820	1.005 (0.987-1.024)	0.583	0.998 (0.976-1.021)	0.885

GLS = global longitudinal strain; GCS = Global circumferential strain; EF = ejection fraction; EDVi = end-diastolic volume index; ESVi = end-systolic volume index; mPAP = mean pulmonary artery pressure. *$p<0.05$

2 Supplementary Figures

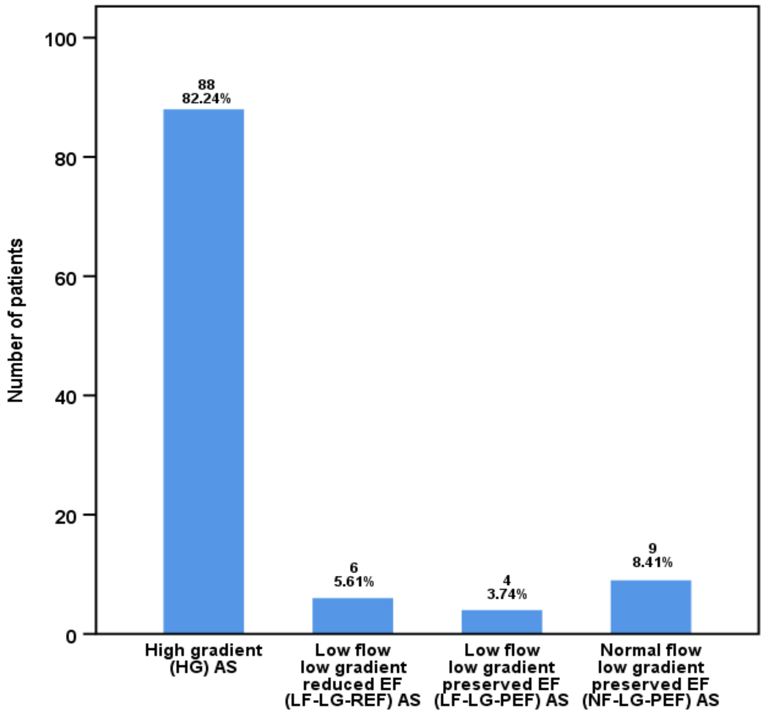

Supplementary Figure 1. Distribution of aortic stenosis (AS) types in the cohort according to Baumgartner et al.(1)

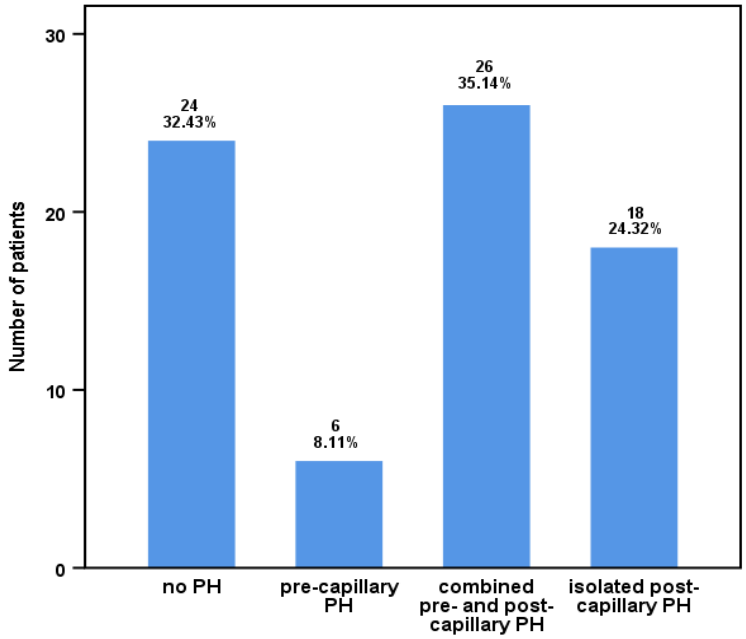

Supplementary Figure 2. Pulmonary hypertension (PH) types according to Simonneau et al.(2) (available in n=74)

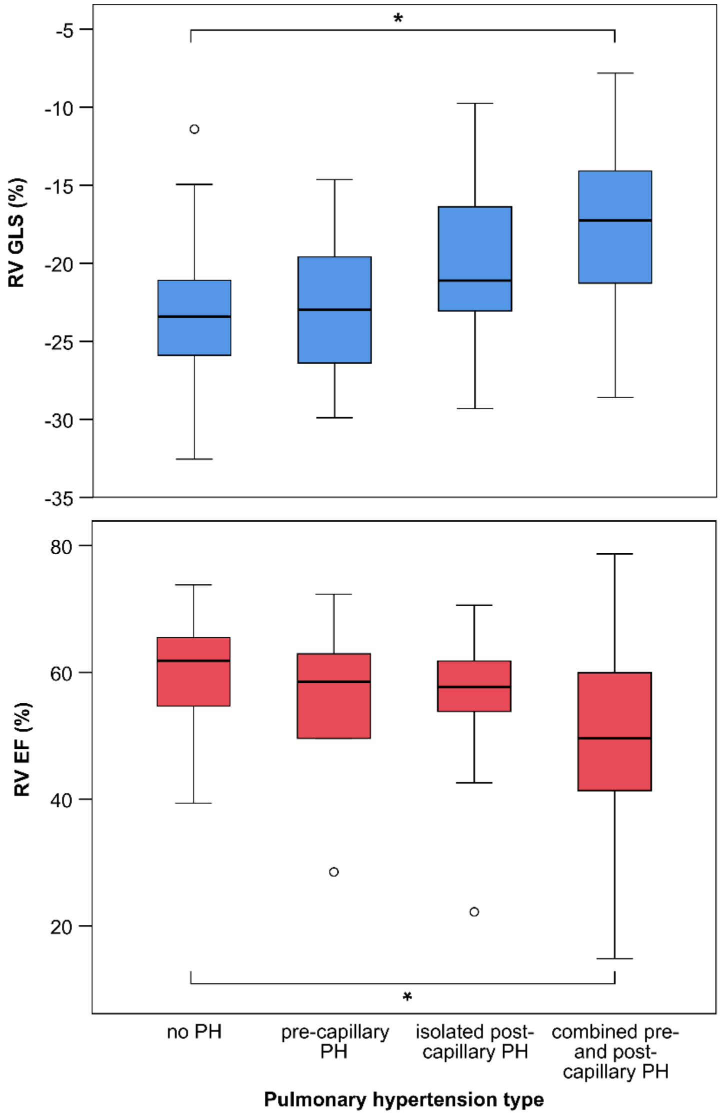

Supplementary Figure 3. Box plots of right ventricular global longitudinal strain (RV GLS) and ejection fraction (RV EF) according to pulmonary hypertension (PH) type (available in n=74). Circles denote outliers (>1.5 times interquartile range above/below upper/lower quartile); *p<0.05 in post hoc analysis (Bonferroni correction).

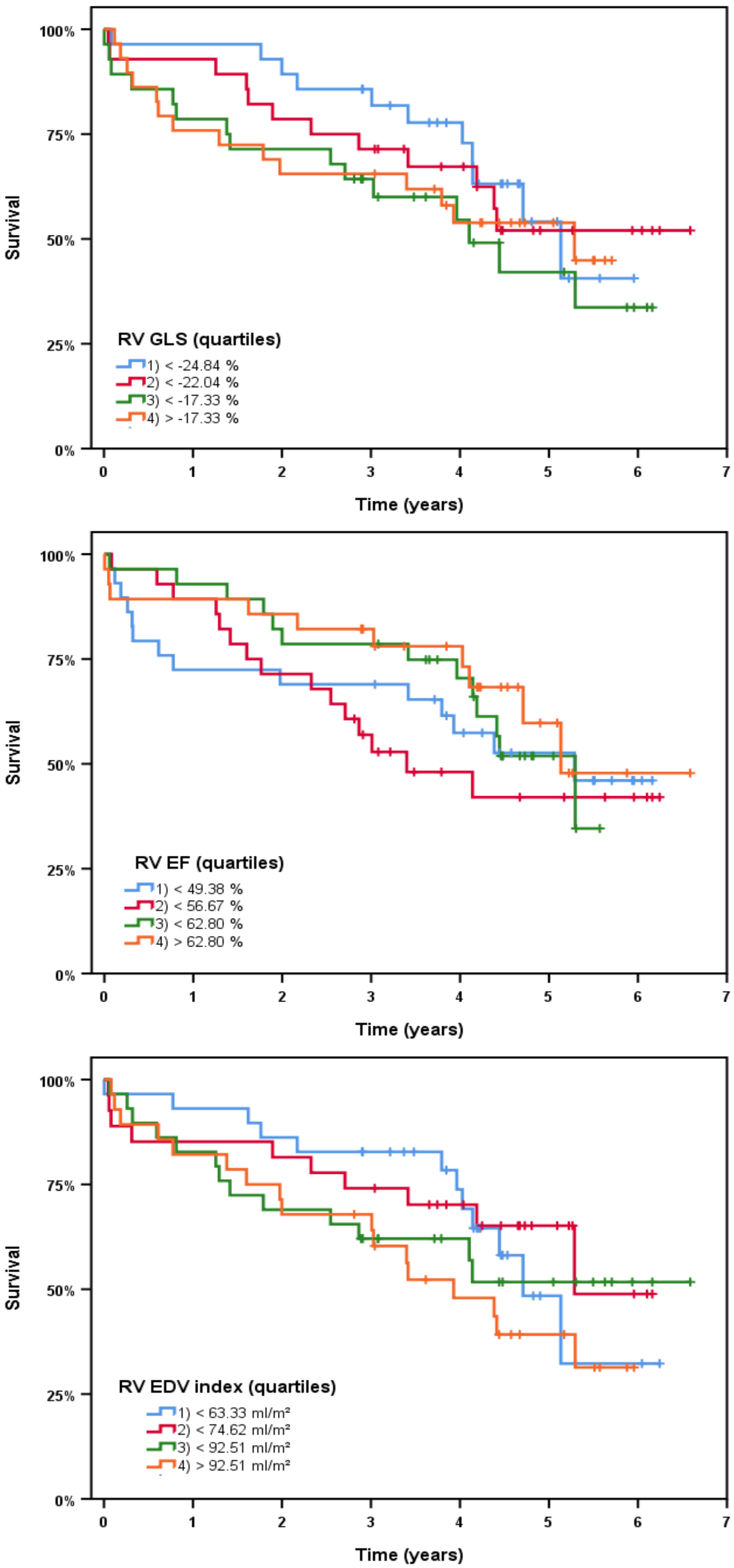

Supplementary Figure 4. Kaplan Meier plots for all-cause mortality and full available follow-up stratified by quartiles of RV and LV parameters. (**Continued on next page**)

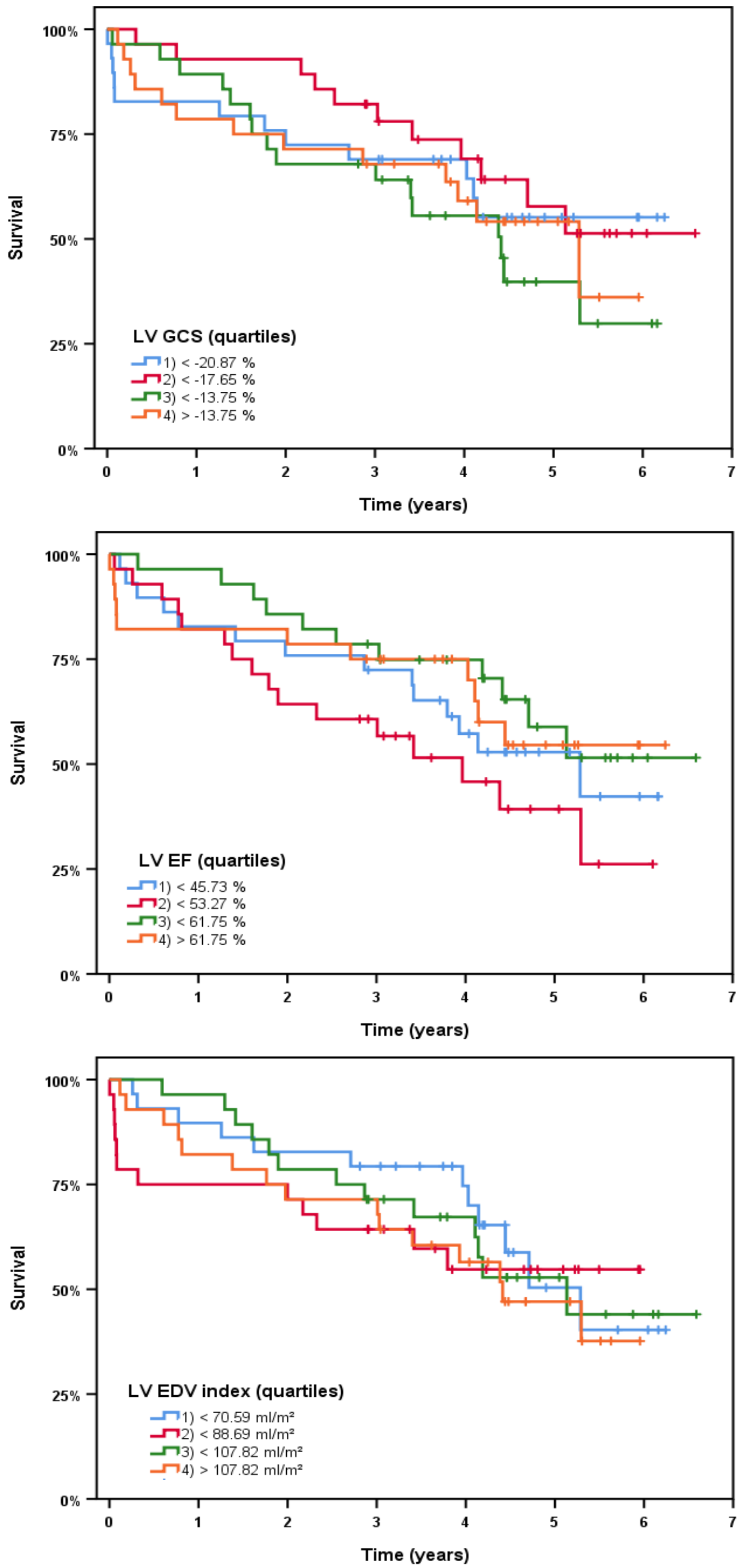

Supplementary Figure 4. (Continued) Kaplan Meier plots for all-cause mortality and full available follow-up stratified by quartiles of RV and LV parameters.

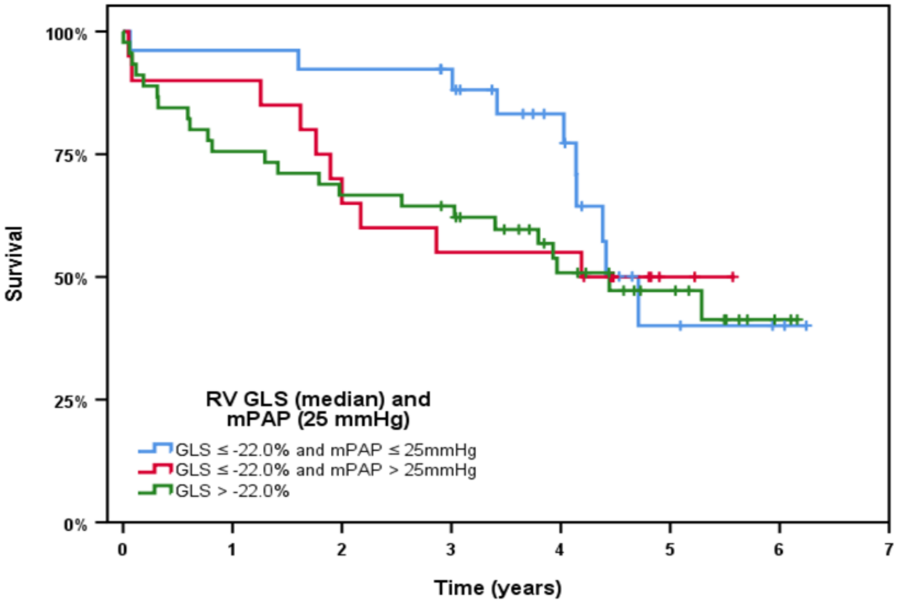

Supplementary Figure 5. Kaplan Meier plot for all-cause mortality according to RV GLS and mPAP. Survival prediction by RV GLS is modified by mPAP.

3 References

1. Baumgartner H, Falk V, Bax JJ, De Bonis M, Hamm C, Holm PJ, et al. 2017 ESC/EACTS Guidelines for the management of valvular heart disease. Eur Heart J. 2017 21;38(36):2739–91.

2. Simonneau G, Montani D, Celermajer DS, Denton CP, Gatzoulis MA, Krowka M, et al. Haemodynamic definitions and updated clinical classification of pulmonary hypertension. Eur Respir J. 2019 Jan;53(1).
